# The Potential of Chlorogenic Acid in Regulating Oxidative Damage, Lipid Metabolism, and Inflammation in Chickens: A Comprehensive Review

**DOI:** 10.3390/vetsci13030267

**Published:** 2026-03-13

**Authors:** Bo Zheng, Xueqing Xiao, Yanli Wang, Dongying Bai, Wenrui Zhen, Fangshen Guo, Bingkun Zhang, Yi Zhang, Yanbo Ma

**Affiliations:** 1 Department of Animal Physiology, College of Animal Science and Technology, Henan University of Science and Technology, Luoyang 471003, China; zhengbo@stu.haust.edu.cn (B.Z.); xueqingxiao@stu.haust.edu.cn (X.X.); wangyanli@stu.haust.edu.cn (Y.W.); 9900427@haust.edu (D.B.); 9906203@haust.edu.cn (W.Z.); 15206405622@163.com (F.G.); 2 Henan International Joint Laboratory of Animal Welfare and Health Breeding, College of Animal Science and Technology, Henan University of Science and Technology, Luoyang 471000, China; 3 State Key Laboratory of Animal Nutrition, Department of Animal Nutrition and Feed Science, College of Animal Science and Technology, China Agricultural University, Beijing 100193, China; bingkunzhang@126.com; 4 Innovative Research Team of Livestock Intelligent Breeding and Equipment, Science & Technology Innovation Center for Completed Set Equipment, Longmen Laboratory, Luoyang 471023, China

**Keywords:** inflammation, chickens, oxidative stress, lipid metabolism, chlorogenic acid

## Abstract

Heat stress, high stocking density, and feed safety risks are common features of modern intensive poultry production and collectively disrupt metabolic homeostasis in chickens. These stressors promote oxidative stress, lipid metabolic dysfunction, and inflammatory activation, ultimately reducing growth performance and overall health. This review summarizes current evidence on the effects of major stressors under intensive farming conditions on lipid metabolic homeostasis, redox balance, and immune regulatory networks, with particular emphasis on the supplementation strategies and physiological effects of chlorogenic acid (CGA) in chickens. Available studies indicate that CGA may regulate lipid metabolic homeostasis through adenosine monophosphate-activated protein kinase (AMPK)-related signaling pathways, enhance antioxidant defenses mediated by nuclear factor erythroid 2 related factor 2 (Nrf2), and suppress inflammatory responses associated with nuclear factor κB (NF-κB), thereby contributing to protective regulation at multiple levels.

## Simple Summary

This review synthesizes available evidence on the relationships among oxidative stress, lipid metabolic dysregulation, and inflammatory responses in chickens under modern intensive production systems, with emphasis on the regulatory role of chlorogenic acid. We discussed how major environmental, nutritional, and management-related stressors, including heat stress, high stocking density, feed contamination, and pathogen challenges, promote reactive oxygen species accumulation, lipid metabolic imbalance, endocrine dysregulation, and immune dysfunction. Furthermore, we summarize evidence showing that chlorogenic acid modulates oxidative status, lipid metabolism, and inflammatory responses through pathways involving adenosine monophosphate-activated protein kinase (AMPK), nuclear factor erythroid 2 related factor 2 (Nrf2), nuclear factor κB (NF-κB), and the gut–liver axis. We also review current dietary supplementation strategies and application approaches of chlorogenic acid in chicken production. Overall, this review summarizes existing evidence on stress-associated metabolic and inflammatory disturbances in chickens and outlines the regulatory effects of chlorogenic acid.

## 1. Introduction

With the continuous growth of the global population and the increasing demand for animal-derived protein, poultry products have become an important and stable source of high-quality protein in the human diet. To meet the expanding market demand, modern poultry production has gradually developed into an intensive system characterized by high stocking density and short production cycles [1,2]. Meanwhile, long-term genetic selection focused on growth rate, feed conversion efficiency, and egg production has substantially improved production efficiency [3,4], but has also increased the metabolic burden of modern chickens and weakened their capacity to maintain homeostasis and adapt to environmental stressors, thereby elevating the risk of metabolism-related health disorders [5,6].

Under commercial production conditions, chickens are typically exposed simultaneously to multiple stressors, including high stocking density, fluctuations in ambient temperature, and unstable feed quality. These external stimuli often act through overlapping molecular signaling pathways, jointly inducing oxidative stress and inflammatory activation and further promoting the development of lipid metabolic disorders [7,8,9,10,11]. Because chickens maintain a relatively high body temperature (approximately 40.6–41.7 °C) and lack efficient heat-dissipation mechanisms, their tolerance to stressful environments is limited [12], making them more susceptible to physiological homeostatic imbalance under intensive production conditions. Accumulating evidence indicates that chronic or repeated stress exposure exacerbates inflammatory damage in key metabolic organs, such as the intestine and liver, and is accompanied by increased mortality and reduced production performance [13,14,15,16,17]. Consequently, stress associated with metabolic dysregulation and inflammatory injury has emerged as a major constraint on the health status and productivity of modern intensive poultry systems.

Notably, oxidative stress, lipid metabolic disorders, and inflammatory responses do not represent independent pathological events. Extensive studies in mammals have demonstrated that these processes are closely interconnected and can form self-amplifying pathological cycles that drive the initiation and progression of multiple metabolic diseases [18,19,20]. Although this knowledge is largely derived from mammalian models, accumulating evidence suggests that similar interactive networks may also exist in poultry [21,22]. However, under intensive production conditions, the coordinated regulatory mechanisms linking lipid metabolism, oxidative stress, and inflammation in chickens remain insufficiently characterized and require further systematic investigation from multi-level perspectives.

In recent years, increasing attention has been directed toward natural bioactive compounds as functional feed additives in poultry nutrition, particularly for their potential roles in alleviating oxidative stress, modulating inflammatory responses, and improving lipid metabolism [23,24,25,26,27]. Chlorogenic acid (CGA) is a water-soluble phenolic acid widely distributed in plants, and numerous studies have demonstrated its antioxidant, anti-inflammatory, and metabolic regulatory activities [28,29,30,31]. These properties suggest that CGA may have potential value in modulating stress-related metabolic disturbances. Therefore, this review aims to systematically integrate available evidence to elucidate the interactive networks among lipid metabolism, oxidative stress, and inflammation in chickens and to clarify the regulatory mechanisms of CGA, thereby providing a clearer theoretical basis for its scientific application as a functional feed additive and a potential alternative to antibiotics in poultry production.

## 2. Major Stressors in Modern Poultry Production Systems

In modern intensive poultry production systems, chickens are chronically exposed to a wide range of external and internal stressors, which often act in an additive and synergistic manner and exert profound effects on growth performance, metabolic homeostasis, and immune function [32,33,34]. At the physical environmental level, abnormal temperatures (including heat stress, cold stress, and temperature fluctuations) [21,35], inadequate humidity and ventilation, deteriorated air quality resulting from the accumulation of ammonia, dust, and carbon dioxide [36], as well as noise and vibration stimuli [37], can interfere with thermoregulation, respiratory function, and neuroendocrine rhythms, thereby imposing sustained physiological burdens. With respect to chemical and contaminant exposure, feed contamination with mycotoxins [38,39], abnormal drinking water quality, and exposure to chemical irritants within poultry houses [40] can impair intestinal and hepatic barrier functions, induce oxidative stress, and weaken host immune defenses.

Meanwhile, stress arising from housing systems and social interactions should not be overlooked. High stocking density, spatial restriction, flock regrouping, and social hierarchy conflicts can readily induce feather pecking, aggression, and fear-related behaviors, thereby exacerbating chronic stress conditions [41,42]. During production management and logistics, procedures such as catching, sorting, and regrouping, transportation, feed and water withdrawal, and pre-slaughter handling often constitute short-term periods of intense stress, further amplifying the risk of physiological and metabolic disturbances [43,44,45,46]. In addition, during early life stages, factors including incubation conditions, chick-processing procedures, post-hatch transportation, adaptation to housing environments, and early feed or water deprivation may exert long-term effects on subsequent growth and stress sensitivity through early life programming mechanisms [47,48]. With continuous genetic selection for enhanced production performance, endogenous metabolic burdens associated with rapid growth, high metabolic rates, and stage-specific production peaks have also emerged as important sources of physiological stress.

## 3. Stress-Induced Systemic Metabolic Dysregulation

### 3.1. Hypothalamic–Pituitary–Adrenal (HPA) Axis and Corticosterone-Mediated Endocrine Responses

Under various environmental stress conditions, chickens rapidly initiate endocrine responses through activation of the HPA axis. Stress perception stimulates the hypothalamus to release corticotropin-releasing hormone (CRH), which subsequently induces the secretion of adrenocorticotropic hormone (ACTH) from the anterior pituitary. ACTH then acts on the adrenal glands to promote the synthesis and release of corticosterone (CORT), the primary glucocorticoid in birds. Elevated CORT concentrations play a central role in coordinating metabolic adaptation to stress by regulating glucose production, lipid mobilization, and immune responses [49,50,51]. Elevated CORT binds to the glucocorticoid receptor (GR), thereby promoting gluconeogenesis, protein catabolism, and lipid mobilization. These metabolic alterations increase mitochondrial substrate flux and electron transport chain load, enhance electron leakage, and consequently induce excessive generation of reactive oxygen species (ROS) and elevated lipid peroxidation, as reflected by increased thio barbituric acid reactive substances (TBARS) and malondialdehyde (MDA) concentrations [52,53].

At the level of metabolic regulation, persistently elevated CORT markedly remodels hepatic energy metabolism. On the one hand, CORT promotes skeletal muscle protein breakdown to provide substrates for gluconeogenesis, thereby meeting the increased energy demands under stress conditions. On the other hand, prolonged CORT exposure enhances lipogenic pathway activity, inducing upregulation of key lipogenic genes, including fatty acid synthase (FASN), acetyl-CoA carboxylase (ACC), and sterol regulatory element-binding protein-1c (SREBP-1c), and is accompanied by pronounced hepatic lipid accumulation [50]. AMP-activated protein kinase (AMPK) is a central sensor of cellular energy status that is activated when the intracellular AMP/ATP ratio increases and functions to maintain metabolic homeostasis. Once activated, AMPK promotes catabolic pathways that generate ATP while suppressing anabolic processes such as fatty acid and cholesterol synthesis [54,55]. In the liver, AMPK activation inhibits key lipogenic regulators including ACC and SREBP-1c, while enhancing fatty acid oxidation [56]. Under chronic stress conditions, sustained elevation of CORT may interfere with AMPK signaling and disrupt lipid metabolic balance, leading to reduced lipid oxidation and increased hepatic lipid accumulation [57,58].

At the immunoregulatory level, chronic elevation of CORT also exerts profound effects on immune homeostasis. Although acute stress can transiently activate innate immunity and acute phase responses, sustained glucocorticoid exposure progressively aggravates immunosuppression [59]. Previous studies have shown that long-term CORT treatment induces marked atrophy of immune organs, such as the bursa of Fabricius, and is accompanied by declines in immune cell number and function [60]. Classical immunological theory further indicates that glucocorticoids inhibit the proliferation and differentiation of adaptive immune cells and interfere with the fine regulation of inflammatory responses [61]. Consequently, under chronic stress conditions, CORT-mediated immunosuppression and persistent low-grade inflammatory activation often coexist, leading to an abnormal immune state characterized by impaired defense capacity and dysregulated inflammatory signaling.

### 3.2. Disruption of Free Radical Homeostasis and Ionic Balance

Under stress conditions, the dynamic balance between antioxidant defense systems and ROS production in chickens is frequently disrupted, leading to a sustained state of oxidative stress [40,62]. As byproducts of normal cellular metabolism, ROS are mainly generated by the mitochondrial respiratory chain, endoplasmic reticulum (ER), peroxisomes, and plasma membrane-associated enzyme systems, and they participate in signal transduction and metabolic regulation under physiological conditions [63]. However, under stressful environments, the rate of ROS generation markedly exceeds cellular scavenging capacity, ultimately inducing oxidative damage and metabolic homeostasis disruption [64,65,66], which may progress to tissue injury and organ dysfunction in severe cases [67,68].

From the perspective of energy metabolism, chickens exhibit relatively high basal metabolic rates and oxidative phosphorylation activity, rendering them more susceptible to abnormal ROS accumulation under production-related stress [69]. Multiple exogenous factors, including environmental stressors, mycotoxin exposure, pathogen infection, and heavy metal residues, together with endogenous factors such as mitochondrial dysfunction, increased fatty acid metabolic load, and amplified inflammatory signaling, act synergistically to drive persistent ROS production [40,70]. Stress-induced enhancement of electron leakage from the mitochondrial electron transport chain and impairment of adenosine triphosphate synthesis efficiency further promote the formation of reactive oxygen species, particularly superoxide anions [71,72,73]. In parallel, nicotinamide adenine dinucleotide phosphate (NAD(P)H) oxidases represent another major enzymatic source of ROS. Their activity can be positively regulated by mitochondrial-derived ROS, and they have been shown to participate in cytosolic ROS amplification cascades in chicken models [74,75,76,77,78].

Sustained oxidative burden not only compromises the structural integrity of lipids, proteins, and deoxyribonucleic acid (DNA) but is also frequently accompanied by disturbances in acid–base balance and ionic homeostasis. Under heat stress, chickens enhance heat dissipation through panting, leading to excessive CO_2_ loss and respiratory alkalosis, which is subsequently followed by renal compensatory responses and secondary metabolic disturbances [79]. During this process, the loss of electrolytes such as Na^+^ and K^+^ exacerbates intracellular instability and weakens transmembrane transport and signal transduction. Meanwhile, inhibition of carbonic anhydrase activity interferes with ion formation and transport, further affecting physiological processes such as calcium deposition and tissue mineralization [80].

The combined effects of ionic imbalance and oxidative damage markedly impair the structural integrity and functional stability of key organelles, including mitochondria and the ER, thereby accelerating cellular metabolic dysregulation and stress responses. Among these processes, disruption of Ca^2+^ homeostasis represents a central hub linking oxidative stress to organelle dysfunction [81]. Under ER stress, large amounts of Ca^2+^ are released from the ER lumen and rapidly taken up by mitochondria within high-Ca^2+^ microdomains formed at mitochondria-associated membranes (MAMs). Excessive Ca^2+^ transfer induces mitochondrial Ca^2+^ overload, leading to mitochondrial dysfunction and cell death [82,83,84]. Ca^2+^ overload triggers the opening of the mitochondrial permeability transition pore (mPTP), disrupts membrane potential stability, and promotes the release of pro-apoptotic factors such as cytochrome c [85]. In addition, excessive Ca^2+^ enhances respiratory chain flux and further amplifies ROS generation, forming a Ca^2+^-ROS positive feedback loop [86].

Overall, multiple environmental stressors induce persistent ROS overload and ionic imbalance, synergistically impair mitochondrial and ER function, and promote Ca^2+^ signaling disruption, energy metabolic dysfunction, and abnormal activation of cellular stress pathways. These alterations provide a critical molecular basis for inflammatory amplification and the development of metabolic disorders.

### 3.3. Coupling Mechanisms Between Lipid Metabolic Imbalance and Oxidative Signaling

Dysregulated lipid metabolism has emerged as an important health concern in modern intensive poultry production systems. The widespread and long-term application of high-energy feeding strategies has led to an increasing prevalence of excessive fat deposition in both broilers and laying hens [87]. Accumulating evidence indicates that lipid metabolic imbalance not only directly impairs chicken health and production performance but also significantly reduces meat quality and product economic value [88,89,90]. In broilers, fat is mainly deposited in non-functional sites, such as the abdominal cavity and subcutaneous tissues, thereby decreasing feed utilization efficiency and increasing processing losses during slaughter [91,92,93,94]. In laying hens, abnormal lipid accumulation is closely associated with the development of fatty liver hemorrhagic syndrome, which can markedly reduce egg production and increase mortality risk in severe cases [95,96].

In chickens, the liver serves as the central organ for lipid metabolic regulation. It converts excess dietary energy into lipids, coordinates fatty acid synthesis and oxidation, and maintains lipoprotein and cholesterol homeostasis. When hepatic function is disrupted by environmental stress, nutritional imbalance, or endocrine disorders, the dynamic balance between lipid synthesis and degradation is readily disturbed. This disturbance leads to excessive lipid accumulation in the liver and abdominal adipose tissue and is accompanied by abnormal alterations in serum triglyceride concentrations, total cholesterol concentrations, and lipoprotein profiles. Such lipid homeostasis imbalance, characterized by hepatic metabolic reprogramming, may represent an important pathological basis for the progressive aggravation of fat deposition under intensive production conditions.

Notably, lipid metabolic disorders are frequently coupled with oxidative stress and inflammatory responses, forming highly interconnected regulatory networks. Studies have shown that high lipid load and stress conditions markedly promote excessive ROS generation, inducing lipid peroxidation, membrane structural damage, and mitochondrial dysfunction, thereby further disrupting energy metabolic homeostasis [97]. Persistent ROS accumulation not only directly impairs lipid metabolism-related enzyme systems but also indirectly regulates lipid synthesis and degradation pathways through the activation of nuclear factor κB (NF-κB) and mitogen-activated protein kinase (MAPK) signaling pathways [98]. These processes continuously amplify lipid metabolic imbalance and promote the progression of abnormal fat deposition.

### 3.4. Stress Driven Activation and Amplification of Inflammatory Signaling Networks

Inflammatory responses represent one of the major limiting factors affecting intestinal barrier function, hepatic metabolic homeostasis, and overall growth performance in chickens. Compared with mammals, chickens rely more heavily on innate immune defenses, whereas the initiation and maturation of adaptive immune responses are relatively delayed, particularly during early developmental stages. This immunological characteristic renders chickens more susceptible to rapid initiation and sustained amplification of inflammatory responses under environmental stress or pathogenic stimulation [99,100,101].

In chickens, Toll-like receptors (TLRs) are widely expressed in the intestine and various immune-related organs [102]. Among these receptors, the Toll-like receptor 4 (TLR4)–myeloid differentiation primary response 88 (MyD88)–NF-κB signaling axis is considered a major pro-inflammatory pathway mediating endotoxin responses and intestinal inflammation [103]. Numerous studies have demonstrated that this pathway is persistently activated under conditions of heat stress, endotoxin exposure, or mixed infections and is accompanied by excessive cytokine release and impairment of intestinal barrier function [104,105,106,107].

Oxidative stress and organ dysfunction provide a critical molecular basis for abnormal amplification of inflammatory signaling. Excessive ROS accumulation enhances the sensitivity of pattern recognition receptors such as TLR4 and activates upstream kinase cascades, thereby promoting the dissociation of NF-κB from inhibitory complexes and its subsequent nuclear translocation. This process drives the transcription of multiple pro-inflammatory cytokines, including tumor necrosis factor-α (TNF-α) and interleukin-6 (IL-6) [108,109]. Studies have shown that under chronic heat stress, hepatic expression levels of TLR4 and NF-κB are markedly elevated in chickens and are accompanied by sustained accumulation of IL-6 and TNF-α [110]. These changes not only induce local tissue inflammation but may also extend to systemic inflammatory responses through the circulatory system.

In addition to the classical NF-κB pathway, the NOD-like receptor family pyrin domain-containing 3 (NLRP3) inflammasome plays a critical role in stress-related inflammatory cascade amplification. Damage-associated molecular patterns (DAMPs) released from damaged mitochondria, including mitochondrial DNA (mtDNA) and cytochrome c, together with excessive ROS accumulation, cooperatively activate the NLRP3 inflammasome complex, leading to caspase-1 activation and promotion of interleukin-1β (IL-1β) maturation and release [111], thereby further reinforcing the inflammatory microenvironment. In addition, MAPK pathways are also involved in transducing stress signals into inflammatory responses. MAPK subtypes, such as p38 MAPK and c-Jun N-terminal kinase (JNK), are persistently activated under various stress conditions and can form coordinated regulatory networks with NF-κB signaling, thereby further amplifying pro-inflammatory gene expression and cellular injury [112].

## 4. Mechanisms by Which Chlorogenic Acid Alleviates Lipid Metabolic Disorders, Oxidative Stress, and Inflammatory Responses

### 4.1. Dietary Sources and Supplementation of Chlorogenic Acid in Chickens

In existing chicken model studies, CGA has mainly been supplemented in the form of purified compounds or plant extracts rich in CGA. To ensure precise dosage control and experimental reproducibility, most studies have used standardized formulations for accurate CGA supplementation. According to the available literature, dietary inclusion levels of CGA in chickens are generally concentrated in the range of several hundred to over one thousand milligrams per kilogram of feed. This dosage range has been applied across different experimental models and stress conditions.

In broiler studies, multiple investigations have systematically evaluated the biological effects of different CGA supplementation levels. For example, Zha et al. supplemented Ross 308 broiler diets with 0, 250, 500, and 1000 mg/kg CGA in a diquat-induced oxidative stress model and found that CGA improved growth performance, enhanced antioxidant enzyme activities, reduced lipid peroxidation, and alleviated hepatic inflammatory injury in broilers [113]. In another high-stocking-density model, Liu et al. supplemented 0.15% CGA and reported improvements in growth performance, jejunal barrier function, antioxidant capacity, and cecal microbial composition in broilers exposed to stocking-density stress [114]. In addition, Wei et al. reported that dietary supplementation with 1.0 g/kg CGA alleviated intestinal injury induced by avian pathogenic Escherichia coli infection and reduced mortality in chickens [28].

In most experiments, CGA is uniformly mixed into basal diets in powder form and subsequently processed into pelleted or mash feeds for consumption. In contrast, studies on CGA supplementation via drinking water remain limited, and this approach is still under exploration. Available evidence indicates that adding 1–2 mL/L CGA to drinking water may improve growth performance and intestinal health-related parameters in broilers [115]. However, its stability and applicability under commercial production conditions require further verification.

**Figure 1:**
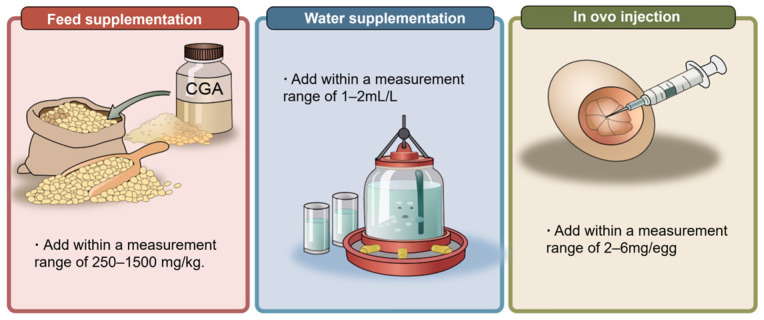
The methods of CGA supplementation in chickens. CGA can be administered to chickens through three main approaches, including dietary supplementation, drinking water supplementation, and in ovo injection. Dietary supplementation involves adding 250–1500 mg/kg of CGA to the feed, enabling stable intake throughout the entire rearing period; drinking water supplementation involves adding 1–2 mL/L of CGA to the drinking water; in ovo injection involves injecting 2–6 mg per egg of CGA into fertilized eggs.

In addition to conventional dietary supplementation, several studies have explored non-traditional CGA administration strategies. For example, Pan et al. supplemented CGA via in ovo injection under heat stress conditions (4 mg per egg) and reported improved post-hatch intestinal antioxidant status, accompanied by upregulation of nuclear factor erythroid 2-related factor 2 (Nrf2)-related genes and epigenetic regulatory factors [116]. This finding suggests potential applications of CGA in early life nutritional interventions in poultry. Furthermore, recent studies have examined combined supplementation strategies involving CGA and other functional additives. For instance, co-supplementation of CGA and baicalin further improved growth performance and immune function in chickens [117]. In addition, combined administration of α-lipoic acid and tea polyphenols alleviated cadmium-induced hepatic injury in Sanhuang chickens [118]. These findings indicate possible synergistic interactions among phenolic acids and antioxidant compounds and provide directions for the development of composite CGA-based supplementation strategies. The main supplementation approaches of CGA in chickens are illustrated in Figure 1.

In addition, to improve its stability and delivery efficiency, various protective delivery systems have been explored in recent years, including liposomes, nanoemulsions, and polysaccharide-coated carriers [119,120,121,122,123]. These delivery strategies may reduce the early degradation of CGA in the gastrointestinal tract and promote its sustained release and absorption in the intestine. Although related research in poultry remains at an early stage, nano-delivery technologies may provide new technical approaches for the application of CGA in precision nutrition and functional feed additives.

### 4.2. Absorption of Chlorogenic Acid in Chickens

The biological effects of CGA largely depend on its absorption efficiency and metabolic transformation in vivo. Although the pharmacokinetics of CGA have been extensively studied in mammals, information regarding its absorption and metabolism in poultry, particularly in chickens, remains limited, and existing studies have mainly focused on its physiological and nutritional regulatory effects. Available evidence suggests that approximately one-third of CGA can be absorbed into systemic circulation through the stomach and small intestine, whereas the remaining portion undergoes microbial metabolism in the intestinal tract [124,125]. A portion of CGA and its hydrolysis products can be absorbed by small intestinal epithelial cells and subsequently transported to the liver via the portal vein for further metabolism. CGA that is not absorbed by the intestinal epithelium, together with its primary hydrolysis product caffeic acid, can enter the cecum and undergo extensive microbial metabolism. Cecal microorganisms degrade CGA through enzymatic hydrolysis and reduction pathways. Certain bacteria, such as *Bifidobacterium animalis*, contain intracellular feruloyl esterase, which can hydrolyze CGA into caffeic acid [126]. In addition, several *Lactobacillus* and *Clostridium* species participate in the further transformation of intermediate metabolites such as caffeic acid and ferulic acid through reactions including decarboxylation, reduction, and side-chain cleavage [127].

In in vitro fermentation studies using human fecal microbiota, CGA is initially hydrolyzed to release caffeic acid and ferulic acid, which are subsequently reduced to various phenolic metabolites, including dihydrocaffeic acid, dihydroferulic acid, and 3-(3′-hydroxyphenyl)propionic acid. These reduced compounds are considered major end-products of CGA metabolism mediated by intestinal microbiota [128]. These metabolites may further participate in biological processes such as antioxidant activity, anti-inflammatory responses, and metabolic regulation in vivo.

### 4.3. Antioxidant Regulatory Mechanisms

The phenolic hydroxyl groups of CGA confer strong free radical-scavenging capacity, enabling the direct neutralization of peroxyl radicals through the formation of stable reaction products [129]. In addition, CGA enhances antioxidant defenses by upregulating endogenous antioxidant systems, thereby strengthening resistance to oxidative damage in poultry [124]. Owing to the abundance of catechol-type ortho-hydroxyl groups in its molecular structure, CGA exhibits strong free radical-scavenging activity [130]. CGA exerts antioxidant effects through direct scavenging of ROS, free radical transfer and hydrogen-donating reactions, as well as metal ion chelation [131]. It can react with hydroxyl radicals and superoxide anions to form relatively stable phenoxyl radicals, thereby interrupting the chain propagation of lipid peroxidation [132]. In addition, CGA can chelate transition metal ions such as Fe^2+^ and Cu^2+^, inhibit Fenton reactions, and further reduce hydroxyl radical generation [133,134,135].

In chicken studies, CGA has been shown to promote the dissociation of Kelch-like ECH-associated protein 1 (Keap1) from Nrf2, facilitating Nrf2 nuclear translocation and binding to antioxidant response element (ARE). This process induces the expression of antioxidant-related genes, including heme oxygenase-1 (HO-1), NAD(P)H: quinone oxidoreductase 1 (NQO1), and glutamate-cysteine ligase modifier subunit (GCLM) [136,137]. In addition, CGA may indirectly activate Nrf2 signaling through autophagy-related mechanisms, thereby alleviating oxidative stress in broilers and improving growth performance and intestinal health [138]. In laying hen models, CGA supplementation under hydrogen peroxide-induced oxidative stress conditions has been reported to increase egg production, reduce hepatic lipid accumulation, and improve mitochondrial function [139]. These findings indicate that the antioxidant activity of CGA is not restricted to the intestine but may provide systemic protection at the whole-body level.

At present, direct evidence regarding the regulation of NAD(P)H oxidases by CGA in chickens remains limited. However, studies in other animal models have shown that CGA can reduce sustained ROS production by inhibiting NAD(P)H oxidase activity [140,141]. Based on available evidence, suppression of NAD(P)H oxidase-mediated ROS generation may represent a complementary mechanism contributing to the antioxidant effects of CGA. Nevertheless, this hypothesis requires further validation through targeted studies in chicken models.

### 4.4. Regulatory Effects on Lipid Metabolism

Available evidence indicates that the regulatory effects of CGA on lipid metabolism in chickens are mainly mediated through two complementary pathways: inhibition of de novo lipogenesis and enhancement of fatty acid transport and β-oxidation. Fatty acid and cholesterol synthesis represent key processes underlying lipid deposition, and de novo lipogenesis is considered an important source of endogenous fat accumulation [142,143]. AMPK, a key sensor of cellular energy status, rapidly suppresses lipogenesis through multiple downstream targets [144,145,146]. Previous studies have shown that CGA activates AMPK signaling and promotes the phosphorylation-dependent inactivation of ACC, thereby reducing malonyl-CoA production, limiting substrate availability for fatty acid synthesis, and suppressing lipid accumulation [147]. In addition, CGA and its related metabolites have been reported to inhibit the cascade from acetyl-CoA to fatty acid synthesis through AMPK-mediated negative regulation [139].

With respect to fatty acid catabolism, studies have demonstrated that CGA enhances the expression of peroxisome proliferator-activated receptor α (PPARα) and its downstream target genes, including carnitine palmitoyltransferase 1A (CPT1A) and acyl-CoA oxidase 1 (ACOX1), thereby increasing β-oxidation capacity [140]. CPT1A serves as the rate-limiting protein for mitochondrial fatty acid transport, whereas ACOX1 is a key enzyme involved in peroxisomal fatty acid oxidation [148]. In laying hen models, adiponectin (ADPN) may serve as an important intermediary through which CGA modulates AMPK signaling. By engaging the ADPN-AMPK signaling axis in laying hen models, this regulatory process may further contribute to PPARα-associated lipid metabolic regulation, thereby synergistically enhancing fatty acid transport and oxidative capacity [30].

It should be noted that the status of lipid metabolism in vivo not only influences physiological metabolism in chickens but may also affect post-slaughter meat quality. Fatty acid composition and the degree of lipid oxidation are key determinants of poultry meat flavor, nutritional value, and storage stability. Previous studies have reported that by regulating pathways associated with lipid synthesis and fatty acid oxidation, CGA can modify the fatty acid composition of muscle tissue and reduce lipid peroxidation to some extent, thereby improving the oxidative stability and shelf life of poultry meat [149,150]. Therefore, CGA may have potential applications in the regulation of poultry meat quality.

### 4.5. Anti-Inflammatory and Immunoregulatory Functions

In recent years, increasing evidence has indicated that CGA exhibits anti-inflammatory potential in poultry and exerts regulatory effects under various stress models [151,152,153,154]. At the early stage of inflammatory signal transduction, CGA has been reported to downregulate the expression of TLR4 and its downstream adaptor MyD88, thereby attenuating the initial amplification of pro-inflammatory signaling and influencing the overall intensity of subsequent cascades [155]. In multiple inflammatory models, CGA supplementation has been consistently associated with reduced expression of NF-κB downstream pro-inflammatory mediators, including IL-6, lipopolysaccharide-induced tumor necrosis factor-α factor (LITAF), and C-X-C motif chemokine ligand 8 (CXCL8) [156].

In addition to the NF-κB pathway, MAPK signaling also functions as an auxiliary and parallel regulatory pathway in stress-related inflammatory responses. Under environmental stress, endotoxin stimulation, and oxidative injury, increased phosphorylation of its major branches, including extracellular signal-regulated kinase 1/2 (ERK1/2), JNK, and p38 MAPK, is commonly associated with enhanced inflammatory activity [104,157]. Limited evidence suggests that CGA may modulate gut microbiota composition and concurrently influence MAPK signaling, which is associated with improvements in growth performance and immune function in broilers [40].

At the tissue level, CGA supplementation is closely associated with improvements in intestinal mucosal barrier structure. Studies have shown that in lipopolysaccharide or heat stress models, CGA supplementation promotes the recovery of tight junction protein expression and is accompanied by improved villus morphology and alleviated mucosal injury [158]. Meanwhile, CGA reduces the accumulation of ROS and lipid peroxidation products such as MDA in intestinal tissues, thereby mitigating secondary tissue damage under inflammatory conditions [154]. At the hepatic level, CGA supplementation has been associated with decreased expression of NF-κB and its downstream inflammatory mediator IL-1β, accompanied by reduced inflammatory cell infiltration and tissue injury, which contributes to the maintenance of hepatic metabolic homeostasis [125].

In recent years, several studies in chicken models have proposed that CGA may interfere with DNA sensing-related innate immune pathways. For example, in a necrotic enteritis-challenged broiler model, CGA was reported to reduce mtDNA leakage and downregulate the activation of the cyclic GMP-AMP synthase (cGAS)–stimulator of interferon genes (STING)–TANK-binding kinase 1 (TBK1) signaling pathway, thereby alleviating intestinal inflammation and tissue injury [159]. Given that the cGAS-STING axis has been confirmed to be functional in chickens [160,161], and that mtDNA release can trigger inflammatory responses in avian models [162,163], this pathway may represent an emerging complementary mechanism underlying the anti-inflammatory effects of CGA. However, current evidence is mainly derived from specific disease models, and its general applicability under different stress or infection conditions requires further investigation.

In addition, CGA has been reported to suppress the expression of cyclooxygenase-2 (COX-2) and reduce the production of inflammatory mediators derived from arachidonic acid metabolism, thereby weakening the amplification of lipid-associated inflammatory signaling [164]. In addition to classical signaling pathways, recent studies have begun to examine the potential role of CGA in the epigenetic regulation of stress-related genes. Epigenetic modifications, including DNA methylation and histone modification, are considered to play important roles in environmental stress adaptation, immune regulation, and the maintenance of metabolic homeostasis [165].

In chicken studies, although direct evidence remains limited, available reports suggest that CGA may influence the expression patterns of antioxidant-related genes by modulating oxidative stress-associated signaling pathways, such as Nrf2 and its downstream antioxidant enzyme system [166]. However, research on the epigenetic regulatory effects of CGA in chickens remains limited, and the specific mechanisms underlying its role in stress adaptation and metabolic regulation require further investigation.

### 4.6. Gut Microbiota and the Gut–Liver Axis in Chlorogenic Acid Regulation

As a natural polyphenol, CGA exhibits certain prebiotic properties, selectively promoting the proliferation of beneficial bacteria and modulating intestinal microbial structure. Studies have shown that the relative abundance of *Lactobacillus* in the cecum is higher in CGA-treated groups than in control groups [114]. In addition, CGA supplementation increases the relative abundance of acid-producing bacteria within the phylum Firmicutes while reducing the abundance of certain potentially harmful bacteria [154].

Changes in gut microbial composition further promote the proliferation of bacteria responsible for the production of short-chain fatty acids (SCFAs). Several studies have reported that CGA supplementation increases the abundance of butyrate-producing bacteria, including members of Lachnospiraceae, Clostridiaceae, and Coriobacteriaceae within the phylum Firmicutes [167]. For example, in an oxidative stress model in laying hens, CGA supplementation significantly increased the abundance of butyrate-producing bacteria such as *Oscillibacter*, *Turicibacter*, *Intestinimonas*, and Butyricicoccaceae in the cecum [154]. Correspondingly, SCFAs, as important microbial metabolites, play key roles in regulating host immune responses and maintaining intestinal homeostasis [168]. SCFAs promote intestinal epithelial cell differentiation and maintain tight junction protein expression, thereby strengthening the intestinal mucosal barrier. Studies have shown that butyrate significantly upregulates the expression of tight junction proteins and mucosal-related genes. For example, sodium butyrate increases the expression of intestinal genes such as Claudin-1, Occludin, zonula occludens-1 (ZO-1), and mucin 2 (MUC2) in chickens and alleviates villus shortening and increased intestinal permeability caused by lipopolysaccharide (LPS) or stress [169]. In chicken models, butyrate has also been shown to inhibit activation of the TLR4/NF-κB signaling pathway, thereby reducing the expression of inflammatory mediators such as TNF-α [170]. This effect is consistent with the inhibitory action of CGA on the TLR4/NF-κB signaling pathway, suggesting that CGA may indirectly enhance host anti-inflammatory capacity by promoting SCFA production.

**Figure 2:**
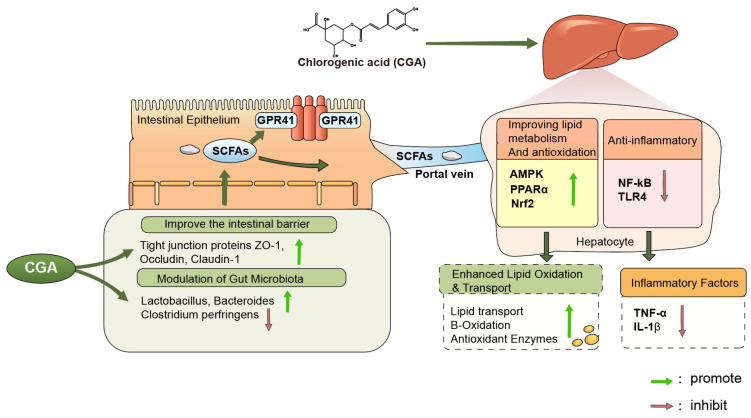
Proposed mechanisms by which CGA regulates lipid metabolism through the gut–liver axis in chickens. CGA modulates gut microbiota composition and promotes the production of SCFAs in the intestine. Increased SCFAs activate GPR41 in intestinal epithelial cells and contribute to the maintenance of intestinal barrier integrity through upregulation of tight junction proteins, including ZO-1, Occludin, and Claudin-1. Microbial metabolites such as SCFAs are transported to the liver through the portal vein and participate in hepatic metabolic regulation. In hepatocytes, CGA influences lipid metabolism and antioxidant responses by regulating signaling pathways including AMPK, PPARα, and Nrf2, while suppressing inflammatory signaling mediated by NF-κB and TLR4. These regulatory processes are associated with enhanced lipid oxidation, reduced inflammatory factor production, and improved metabolic homeostasis. Green arrows indicate promotion, whereas red arrows indicate inhibition.

Through the gut–liver axis, intestinally derived metabolites such as SCFAs can enter the liver via the portal vein and participate in hepatic metabolic regulation. SCFAs, particularly butyrate and propionate, participate in energy metabolism regulation and promote lipid oxidation by activating G protein-coupled receptor 41 (GPR41) and related signaling pathways [158,171]. In addition, butyrate regulates the AMPK/PPARα signaling pathway, promoting fatty acid oxidation and improving lipid metabolic status [172]. In poultry, butyrate can also activate the Nrf2 antioxidant pathway, increasing the expression of antioxidant genes such as HO-1 and NQO1, thereby enhancing hepatic antioxidant capacity [173]. Therefore, CGA may contribute to maintaining chicken health by modulating gut microbial composition, promoting SCFA production, improving intestinal barrier function, and regulating gut–liver axis signaling, thereby coordinately influencing inflammatory responses, oxidative stress, and lipid metabolic homeostasis. These interactions are illustrated in Figure 2.

### 4.7. Multi-Target Coordinated Regulatory Network Model of Chlorogenic Acid

AMPK functions as a metabolic switch that senses cellular energy status and is activated by phosphorylation in response to an increased adenosine triphosphate (ATP) ratio. Once activated, AMPK suppresses lipid synthesis and promotes fatty acid β-oxidation. By activating AMPK signaling, CGA has been shown to alleviate lipid metabolic disorders in the liver and muscle of chickens [30]. In addition, CGA activates the Nrf2-ARE signaling pathway, promotes the transcription of antioxidant genes, and suppresses NF-κB-mediated inflammatory signaling, thereby reducing the release of pro-inflammatory cytokines [174]. These findings indicate that the dual antioxidant and anti-inflammatory activities of CGA not only prevent lipid peroxidation but also indirectly optimize lipid metabolic function by improving the cellular microenvironment.

Through the coordinated regulation of key signaling pathways, including AMPK, Nrf2, and NF-κB, CGA integrates multiple functional modules related to lipid metabolic regulation, antioxidant defense, and inflammatory suppression, thereby establishing a multi-level regulatory network. Within this regulatory framework, ROS act as central mediators linking lipid metabolic dysfunction, oxidative stress, and inflammatory signaling activation. Persistent ROS overproduction disrupts metabolic homeostasis and induces abnormal lipid deposition, aggravated oxidative damage, and inflammatory imbalance.

**Figure 3:**
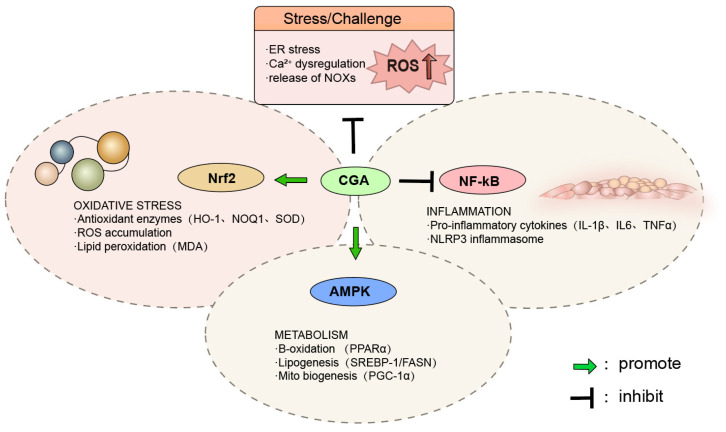
Integrated regulatory network of chlorogenic acid in stress-induced oxidative stress, metabolic dysfunction, and inflammatory responses in chickens. Environmental and physiological stressors induce ER stress, Ca^2+^ dysregulation, and excessive activation of NAD(P)H oxidases, leading to increased ROS production. ROS overaccumulation promotes oxidative damage, lipid peroxidation, metabolic disturbance, and inflammatory activation. CGA exerts regulatory effects by activating Nrf2-mediated antioxidant defenses, thereby upregulating antioxidant enzymes and reducing oxidative stress. Meanwhile, CGA suppresses NF-κB-dependent inflammatory signaling and inhibits pro-inflammatory cytokine production and NLRP3 inflammasome activation. In parallel, CGA activates AMPK signaling, promoting fatty acid β-oxidation, inhibiting lipogenesis, and supporting mitochondrial biogenesis. Through coordinated regulation of oxidative, metabolic, and inflammatory pathways, CGA contributes to the maintenance of cellular homeostasis under stress conditions. Green arrows indicate promotion, whereas T-bars indicate inhibition.

Based on available evidence, CGA may promote fatty acid oxidation and catabolism and inhibit lipid synthesis through activation of AMPK and its downstream PPARα signaling. In parallel, CGA enhances antioxidant enzyme activity by activating Nrf2 signaling, thereby improving ROS-scavenging capacity. Meanwhile, suppression of NF-κB signaling reduces pro-inflammatory gene expression and attenuates inflammatory responses. The coordinated regulation of these pathways enables CGA to partially restore the dynamic balance among lipid metabolism, redox homeostasis, and inflammatory responses. The coordinated regulatory network of chlorogenic acid involving oxidative stress, inflammation, and lipid metabolism in chickens is illustrated in Figure 3.

## 5. Perspectives: Future Directions for Molecular Mechanism Research

In summary, as a natural bioactive compound with multi-target regulatory properties, CGA contributes to the maintenance of metabolic homeostasis and improvement of production performance in chickens through multiple mechanisms, including restoration of lipid metabolic balance, enhancement of antioxidant defenses, and suppression of inflammatory signaling.

Looking ahead, the integration of multi-omics approaches, precision nutrition modeling, and advanced formulation strategies may provide new theoretical foundations and practical pathways for promoting poultry health and ensuring the production of high-quality poultry products.

## Data Availability

No new data were created or analyzed in this study.

## Abbreviations

The following abbreviations are used in this manuscript:

CGA	Chlorogenic acid
CRH	Corticotropin-releasing hormone
ACTH	Adrenocorticotropic hormone
GR	Glucocorticoid receptor
MDA	Malondialdehyde
TBARS	Thio barbituric acid reactive substances
FASN	Fatty acid synthase
ACC	Acetyl-CoA carboxylase
SREBP-1c	Sterol regulatory element-binding protein-1c
AMPK	Adenosine monophosphate-activated protein kinase
CORT	Corticosterone
NAD(P)H	Nicotinamide adenine dinucleotide phosphate
DNA	Deoxyribonucleic acid
ER	Endoplasmic reticulum
MAMs	Mitochondria-associated membranes
MAPK	Mitogen-activated protein kinase
TNF-α	Tumor necrosis factor-α
IL-6	Interleukin-6
TLRs	Toll-like receptors
TLR4	Toll-like receptor 4
MyD88	Myeloid differentiation primary response 88
NLRP3	NOD-like receptor family pyrin domain-containing 3
DAMPs	Damage-associated molecular patterns
mtDNA	Mitochondrial DNA
JNK	C-Jun N-terminal kinase
Keap1	Kelch-like ECH-associated protein 1
ARE	Antioxidant response element
HO-1	Heme oxygenase-1
Nrf2	Nuclear factor erythroid 2 related factor 2
NQO1	NAD(P)H: quinone oxidoreductase 1
GCLM	Glutamate-cysteine ligase modifier subunit
PPARα	Peroxisome proliferator-activated receptor α
CPT1A	Carnitine palmitoyltransferase 1A
ACOX1	Acyl-CoA oxidase 1
ADPN	Adiponectin
SCFAs	Short chain fatty acids
ZO-1	Zonula occludens-1
MUC2	Mucin 2
LPS	Lipopolysaccharide
GPR41	G protein-coupled receptor 41
LITAF	Lipopolysaccharide-induced tumor necrosis factor-α factor
CXCL8	C-X-C motif chemokine ligand 8
ERK1/2	Extracellular signal-regulated kinase 1/2
IL-1β	Interleukin-1β
cGAS	Cyclic GMP–AMP synthase
STING	Stimulator of interferon genes
TBK1	TANK-binding kinase 1
COX-2	Cyclooxygenase-2
ATP	Adenosine triphosphate
